# *Kluyveromyces marxianus* and *Saccharomyces boulardii* Induce Distinct Levels of Dendritic Cell Cytokine Secretion and Significantly Different T Cell Responses *In Vitro*

**DOI:** 10.1371/journal.pone.0167410

**Published:** 2016-11-29

**Authors:** Ida M. Smith, Adam Baker, Jeffrey E. Christensen, Teun Boekhout, Hanne Frøkiær, Nils Arneborg, Lene Jespersen

**Affiliations:** 1 Health & Nutrition Division Discovery, Chr. Hansen A/S, Hørsholm, Denmark; 2 Department of Food Science, University of Copenhagen, Frederiksberg, Denmark; 3 Institute of Metabolic and Cardiovascular Disease, French Institute of Health and Medical Research (INSERM), Toulouse, France; 4 CBS-KNAW Fungal Biodiversity Centre, Utrecht, The Netherlands; 5 Department of Veterinary Disease Biology, University of Copenhagen, Frederiksberg, Denmark; Leibniz Institute for Natural Products Research and Infection Biology- Hans Knoell Institute, GERMANY

## Abstract

Interactions between members of the intestinal microbiota and the mucosal immune system can significantly impact human health, and in this context, fungi and food-related yeasts are known to influence intestinal inflammation through direct interactions with specialized immune cells *in vivo*. The aim of the present study was to characterize the immune modulating properties of the food-related yeast *Kluyveromyces marxianus* in terms of adaptive immune responses indicating inflammation versus tolerance and to explore the mechanisms behind the observed responses. Benchmarking against a *Saccharomyces boulardii* strain with probiotic effects documented in clinical trials, we evaluated the ability of *K*. *marxianus* to modulate human dendritic cell (DC) function *in vitro*. Further, we assessed yeast induced DC modulation of naive T cells toward effector responses dominated by secretion of IFNγ and IL-17 versus induction of a T_reg_ response characterized by robust IL-10 secretion. In addition, we blocked relevant DC surface receptors and investigated the stimulating properties of β-glucan containing yeast cell wall extracts. *K*. *marxianus* and *S*. *boulardii* induced distinct levels of DC cytokine secretion, primarily driven by Dectin-1 recognition of β-glucan components in their cell walls. Upon co-incubation of yeast exposed DCs and naive T cells, *S*. *boulardii* induced a potent IFNγ response indicating T_H_1 mobilization. In contrast, *K*. *marxianus* induced a response dominated by Foxp3^+^ T_reg_ cells, a characteristic that may benefit human health in conditions characterized by excessive inflammation and positions *K*. *marxianus* as a strong candidate for further development as a novel yeast probiotic.

## Introduction

Our gastrointestinal tract contains an overwhelming number of living microorganisms with an increasingly recognized impact on human health[1]. The ability to effectively protect against invading species while maintaining tolerance to commensals and avoiding destructive inflammatory responses to harmless luminal substances is a key feature of the intestinal immune system[2]. In this context, dendritic cells (DCs) present in the mucosal-associated lymphoid tissues lining the human gut are central players involved in microbial sensing and shaping of appropriate adaptive immune responses.

While most studies of microbiota composition have focused solely on the prokaryotic component, communities of eukaryotic microorganisms are present in the mammalian gut[3], and commensal fungi have been found to influence hosts’ susceptibility to colitis[4]. In addition, food-related yeasts and live microorganisms administered as dietary supplements have the potential to impact human health through interactions with intestinal immune cells. Specifically, *Saccharomyces boulardii* (taxonomically acknowledged as belonging to the *S*. *cerevisiae* species[5] but in the following text referred to as *S*. *boulardii*) has shown a positive impact on disease outcome in clinical studies of inflammatory bowel diseases such as Crohn’s disease and ulcerative colitis[6], indicating an ability of *S*. *boulardii* to influence human immune responses underlying intestinal inflammation. The non-*Saccharomyces* yeast species *Kluyveromyces marxianus* comprises food-related yeasts typically isolated from fermented dairy products[7], and the generally non-pathogenic nature of this species is reflected by the fact that *K*. *marxianus* is included in the European Food Safety Authority list of approved microorganisms with qualified presumption of safety (QPS) status[8]. Further, *K*. *marxianus* has been found to engage human immune cells *in vitro*[9, 10] and is of interest as a potential candidate for development of novel yeast probiotics.

DC recognition of microorganisms relies on a wide variety of pattern recognition receptors (PRRs) expressed on the cell surface interacting with specific conserved microbial structures. Yeast recognition is primarily dependent on toll-like receptor (TLR) 2, which recognizes the yeast cell wall preparation zymosan[11, 12], and a number of PRRs known as C-type lectin receptors recognizing specific components of the yeast cell wall[13]. Specifically, the outer mannan layer is recognized by the mannose receptor as well as the DC-specific ICAM-3 grabbing non-integrin (DC-SIGN), while the underlying network of branched β-glucans is recognized by Dectin-1[13–16]. Ligand binding initiates intracellular signaling events in DCs, microbial uptake by phagocytosis, and DC maturation. This process involves modulation of co-stimulatory molecules and changes in chemokine receptor expression as well as in cytokine secretion, reflecting a DC phenotype ready for migration and antigen presentation to naive T cells in draining lymph nodes.

Microbial recognition results in DC secretion of chemokines and cytokines with distinct inflammatory effects. For example, DC secretion of IL-12 promotes expansion of the IFNγ secreting T_H_1 cell subset, whereas DC secretion of IL-1β and IL-6 contributes to a response dominated by the IL-17 secreting T_H_17 subpopulation[17]. Conversely, conditioning DCs toward a predominantly IL-10 secreting phenotype promotes IL-10 secreting Foxp3^+^ regulatory T cells (T_reg_) contributing to intestinal tolerance[2, 18, 19]. In this context, microorganisms with the ability to direct expansion of the T_reg_ subset promote tolerance to the intestinal microbiota and have been found to alleviate symptoms in several conditions characterized by excessive inflammation[20–27].

Microbial cell wall components such as fungal β-glucans are known potentiators of innate immunity, a property that has been explored for antitumor targeting[28–30]. In addition, yeast β-glucans and Dectin-1 signaling have been implicated in protection from type 1 diabetes as well as experimentally induced colitis in rodent models[4, 31, 32], thus representing microbial modulation of host immune responses without administration of live microorganisms.

The aim of the present study was to characterize the immune cell modulating properties of *K*. *marxianus* in terms of adaptive immune responses indicating inflammation versus tolerance. Benchmarking against the established yeast probiotic *S*. *boulardii*, we evaluated the ability of *K*. *marxianus* to modulate human DC function *in vitro*. Further, we evaluated yeast induced DC modulation of naive T cells toward effector responses dominated by IFNγ secreting T_H_1 cells and IL-17 secreting T_H_17 cells versus induction of a T_reg_ response characterized by high levels of IL-10 secretion. In addition, we explored the mechanisms behind the observed responses by blocking relevant DC surface receptors and investigating the immune cell stimulating properties of β-glucan containing yeast cell wall extracts.

## Materials and Methods

### Yeast strains and growth conditions

*Kluyveromyces marxianus* CBS1553 was obtained from CBS-KNAW Fungal Biodiversity Centre (CBS), The Netherlands. *S*. *boulardii* (Ultra-Levure) was obtained from the dietary supplement Ultra-Levure capsules, lot no 7930 (Biocodex, France). Strain identity was verified by DNA sequencing of the D1/D2 domain (NL1/NL4 primers)[33]. Strains were cultured in YPD media (0.5% yeast extract, 1% peptone, 1.1% D-glucose) at 30°C under aerobic conditions. Early stationary growth phase yeast cultures were harvested by centrifugation, washed twice with DC media (RPMI 1640 supplemented with 10 mM HEPES (Sigma-Aldrich, Schnelldorf, Germany) and 50 μM 2-ME (Sigma-Aldrich, Schnelldorf, Germany)), OD adjusted in DC media containing 10% glycerol, and cryopreserved at -80°C until time of DC stimulation. Upon thawing at ambient temperature, viability of yeast cultures was verified by staining with propidium iodide and enumeration of intact yeast cells by flow cytometry. In addition, the cytokine inducing properties of cryopreserved yeast and fresh yeast preparations were compared during the development of the experimental setup. Results showed that cryopreserved and fresh yeast (including among others *S*. *boulardii* and *K*. *marxianus*) induced very similar levels of DC cytokine secretion (IL-12, IL-10, IL-6, and IL-1β).

### Preparation of yeast cell wall extracts

Cell wall extracts of *K*. *marxianus* CBS1553 and *S*. *boulardii* (Ultra-Levure) were prepared according to de Groot *et al*. 2004[34]. Early stationary growth phase yeast cultures were harvested by centrifugation, washed with cold, sterile H_2_O and with 10 mM Tris-HCl, pH 7.5, and resuspended in 10 mM Tris-HCl, pH 7.5 at 1×10^7^ cells/μL. Yeast cells were disintegrated using 250–300 μm glass beads (Sigma-Aldrich, St. Louis, MO, USA) in the presence of a protease inhibitor cocktail (Sigma-Aldrich, St. Louis, MO, USA). Yeast cell disintegration was performed in small volumes (1 mL) by brief, vigorous shaking (30 Hz, 6 min) on a Retsch MM200 instrument (F. Kurt Retsch GmbH, Haan, Germany). To remove non-covalently linked proteins and intracellular contaminants, isolated yeast cell walls were washed extensively with 1 M NaCl and extracted twice for 5 min at 99°C with a detergent solution (50 mM Tris-HCl, pH 7.8, containing 2% SDS, 100 mM Na-EDTA, and 40 mM 2-ME (all from Sigma-Aldrich, St. Louis, MO, USA)). Finally, yeast cell wall extracts were washed three times with sterile H_2_O, resuspended in sterile H_2_O, and stored at -20°C until time of DC stimulation. Absence of culturable yeast cells in the final yeast cell wall extracts was determined by colony counts after 48 h incubation of YPD agar plates at 30°C.

### Monocyte-derived DC generation

Immature monocyte-derived DCs were generated *in vitro* by a 6 day procedure as described by Zeuthen *et al*. 2008[35]. Human buffy coats from healthy donors were supplied by Department of Clinical Immunology at Copenhagen University Hospital, Copenhagen, Denmark. Use of human samples with no identifying information was approved by The National Committee on Health Research and the Danish Society for Clinical Immunology, and all donors gave informed written consent upon donation. Briefly, human PBMCs were obtained from buffy coats by density gradient centrifugation using Ficoll-Paque PLUS (GE Healthcare, Freiburg, Germany). Monocytes were isolated by positive selection for CD14 using magnetic-activated cell sorting with CD14 microbeads (Miltenyi Biotec, Bergisch Gladbach, Germany) and cultured at a density of 2 x 10^6^ cells/mL in complete DC media (RPMI 1640 supplemented with 10 mM HEPES (Sigma-Aldrich, Schnelldorf, Germany), 50 μM 2-ME (Sigma-Aldrich, Schnelldorf, Germany), 2 mM L-glutamine (Life Technologies Ltd, Paisley, UK), 10% heat-inactivated fetal bovine serum (Invitrogen, Paisley, UK), 100 U/mL penicillin (Biological Industries, Kibbutz Beit-Haemek, Israel), and 100 μg/mL streptomycin (Biological Industries, Kibbutz Beit-Haemek, Israel)) containing 30 ng/mL human recombinant IL-4 and 20 ng/mL human recombinant GM-CSF (both from Sigma-Aldrich, Saint Louis, MO, USA) at 37°C, 5% CO_2_. Fresh complete DC media containing full doses of IL-4 and GM-CSF was added after three days of culture. At day 6, differentiation to immature DCs was verified by surface marker expression analysis (CD11c >90% expression; CD1a >75% expression).

### DC stimulation

Immature DCs were resuspended in fresh complete DC media containing no antibiotics, seeded in 96-well plates at 1 × 10^5^ cells/well, and allowed to acclimate at 37°C, 5% CO_2_, for at least one hour before stimulation. DC stimulation using thawed yeast strains was performed at a yeast:DC ratio of 10:1 (MOI 10). DC stimulation using thawed yeast cell wall extracts was performed with extract material prepared from a number of yeast cells corresponding to a yeast:DC ratio of 10:1 (MOI 10). Where indicated, DCs were pre-incubated for 30 min with 20 μM cytochalasin D derived from *Zygosporium mansonii* (Sigma-Aldrich, Saint Louis, MO, USA), 1 μg/mL monoclonal blocking antibodies specific for human Dectin-1/CLEC7A (clone 259931), TLR2 (clone 383936), or DC-SIGN/CD209 (clone 120507), or a nonspecific isotype matched control antibody (all from R&D Systems, Oxon, UK). Stimulated DCs were incubated for 20 h at 37°C, 5% CO_2_, as time-course experiments had shown a 20 h stimulation time to result in quantifiable levels of all cytokines of interest. After 20 h stimulation, DCs were stained for flow cytometric analysis of surface molecule expression or transferred to a 96-well plate for naive T cell co-incubation, and DC supernatants were sterile filtered through a 0.2 μm AcroPrep Advance 96-well filter plate (Pall Corporation, Ann Arbor, MI, USA) and stored at -80°C until time of cytokine quantification.

### DC co-incubation with autologous naive T cells

Autologous, naive CD45RA^+^CD45RO^-^ T cells were isolated from human PBMCs by negative selection using the Naive CD4+ T Cell Isolation Kit II (Miltenyi Biotec, Lund, Sweden) and resuspended in fresh complete DC media at a density of 2 × 10^5^ cells/mL. Co-incubation of yeast stimulated DCs (i.e. DCs that had been pre-exposed to yeast as described under 'DC stimulation') and autologous, naive T cells was performed in 96-well plates at a DC:T cell ratio of 1:20 by combining 2 × 10^4^ DCs with 4 × 10^5^ naive T cells, followed by incubation at 37°C, 5% CO_2_ for 3 days. This co-incubation time was chosen based on time-course data showing equivalent levels of cytokine secretion following co-incubation for 3, 5, and 7 days (data not shown). Where indicated, yeast stimulated DCs were pre-incubated for 30 min with 10 μg/mL monoclonal neutralization antibodies specific for human IL-12p40/p70 (clone C8.6) (BD Biosciences, Temse, Belgium) or TGFβ (clone 1D11) (R&D Systems, Oxon, UK), or appropriate nonspecific isotype matched control antibodies. After 3 days co-incubation, cells were stained for flow cytometric analysis of T_reg_ subset expansion and cell culture supernatants were sterile filtered through a 0.2 μm AcroPrep Advance 96-well filter plate (Pall Corporation, Ann Arbor, MI, USA) and stored at -80°C until time of cytokine quantification.

### DC staining for expression of co-stimulatory molecules and chemokine receptors

Immediately following 20 h stimulation time, DCs were collected, centrifuged at 200 × *g* for 5 min, and resuspended in cold PBS containing 2% BSA. Staining was performed using the following monoclonal antibodies: FITC-conjugated anti-CD80 (clone L307.4), FITC-conjugated anti-CD86 (clone 2331), APC-conjugated anti-CCR6 (clone 11A9), FITC-conjugated anti-CCR7 (clone 150503), and appropriate nonspecific isotype matched controls (all from BD Biosciences, Erembodegem, Belgium). DCs were incubated with 1 μg/mL monoclonal antibodies for 30 min on ice protected from light, followed by repeated wash steps using 1 mL cold PBS 2% BSA. Finally, DCs were resuspended in PBS 2% BSA and kept on ice until flow cytometric analysis. Samples were acquired on an LSRFortessa flow cytometer (BD Biosciences, San Jose, CA, USA) using FACSDiva software (BD Biosciences, San Jose, CA, USA).

### T cell staining for quantification of T_reg_ subset

Following 3 days co-incubation of yeast stimulated DCs and naive T cells, cells were washed using cold CellWash (BD Biosciences, Erembodegem, Belgium) and resuspended in cold Stain Buffer (BD Biosciences, Erembodegem, Belgium). For surface staining, cells were incubated with PE-Cy7-conjugated anti-CD4 (clone SK3) and PerCP-Cy5.5-conjugated anti-CD25 (clone M-A251) or appropriate nonspecific isotype matched controls (all from BD Biosciences, Erembodegem, Belgium) for 30 min on ice protected from light. For intracellular staining, surface-stained cells were fixed and permeabilized using Human Foxp3 Buffer Set (BD Biosciences, Erembodegem, Belgium), incubated with PE-conjugated anti-Foxp3 (clone 259D/C7) or an appropriate nonspecific isotype matched control (both from BD Biosciences, Erembodegem, Belgium), and washed twice before analysis. Stained cells were acquired on an LSRFortessa flow cytometer (BD Biosciences, San Jose, CA, USA) using FACSDiva software (BD Biosciences, San Jose, CA, USA). Specific gates and quadrants were defined based on background staining of isotype controls. At least 10,000 cells were analyzed for each sample.

### Cytokine quantification

Secreted levels of IL-12p70, IL-1β, IL-6, IL-10, IFNγ, and IL-17 were quantified by Cytometric Bead Array Flex Sets (BD Biosciences, Erembodegem, Belgium) according to the manufacturer’s instructions. Briefly, fluorescent beads coated with monoclonal capture antibodies were mixed with PE-conjugated detection antibodies and recombinant standards or test samples and allowed to form sandwich complexes during subsequent incubations protected from light. After repeated wash steps, samples were acquired on an LSRFortessa flow cytometer (BD Biosciences, San Jose, CA, USA) and data analysis was performed using the FCAP Array 3 software (BD Biosciences, San Jose, CA, USA). Detection limits for individual cytokines were as follows: 0.6 pg/mL IL-12, 2.3 pg/mL IL-1β, 1.6 pg/mL IL-6, 0.13 pg/mL IL-10, 1.8 pg/mL IFNγ, and 0.3 pg/mL IL-17.

### Statistical analysis

Data are expressed as mean ± SEM. Statistical analyses, including one-way and two-way ANOVA with Tukey's test for multiple comparisons, were performed using GraphPad Prism 6 (GraphPad Software, La Jolla, USA). Statistically significant results are indicated in figures as follows: *, P<0.05; **, P<0.01; ***, P<0.001; ns, not significant.

## Results

### *K*. *marxianus* and *S*. *boulardii* induce distinct patterns of human DC cytokine secretion and maturation

**Fig 1:**
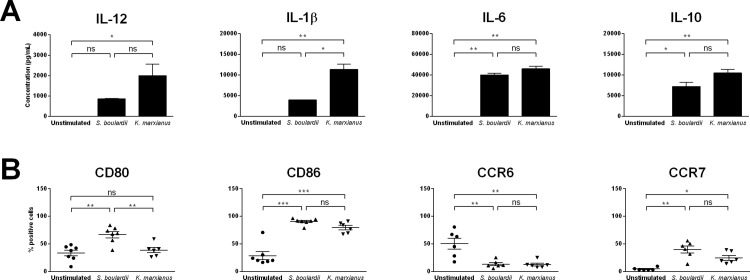
*K*. *marxianus* and *S*. *boulardii* induce distinct DC modulation. **A.** Levels of IL-12, IL-1β, IL-6, and IL-10 secreted by human monocyte-derived DCs following 20 h incubation with DC media (unstimulated), a probiotic reference strain (*S*. *boulardii*), or *K*. *marxianus* at a yeast:DC ratio of 10:1 (MOI 10). Data are representative of at least five independent experiments, error bars represent SEM. **B.** Levels of DC expression of CD80, CD86, CCR6, and CCR7 following 20 h incubation with DC media (unstimulated), a probiotic reference strain (*S*. *boulardii*), or *K*. *marxianus* at a yeast:DC ratio of 10:1 (MOI 10). Data are expressed as mean ± SEM of at least five independent experiments (five donors). One-way ANOVA, Tukey’s multiple comparisons test, indicating significant differences as follows: *, P<0.05; **, P<0.01; ***, P<0.001; ns, not significant.

Based on the fact that commensal fungi and food-related yeasts are known to influence intestinal inflammation through direct interactions with specialized immune cells *in vivo*, we evaluated the ability of *K*. *marxianus* to modulate the function of human DCs *in vitro*. As a point of reference, we included a *S*. *boulardii* strain with probiotic effects documented in clinical trials[6]. Human monocyte-derived DCs were exposed to each yeast strain at a 10:1 yeast:DC ratio (MOI 10) and yeast induced responses were evaluated after 20 h stimulation, as time-course experiments had shown a 20 h stimulation time to result in quantifiable levels of all cytokines of interest (data not shown). As expected, *S*. *boulardii* engaged human DCs and induced robust secretion of IL-12, IL-1β, IL-6, and IL-10 (Fig 1A). Similarly, *K*. *marxianus* induced statistically significant DC secretion of all four cytokines, confirming previous results[10]. While the levels of DC IL-12 and IL-1β secretion appeared to be significantly stronger in response to stimulation with *K*. *marxianus* compared to *S*. *boulardii*, the two yeasts induced similar levels of IL-6 and IL-10 secretion in DCs. In addition, both yeasts induced DC maturation, as assessed by expression levels of co-stimulatory molecules and chemokine receptors (Fig 1B). Specifically, *S*. *boulardii* induced elevated levels of CD80 as well as CD86 expression, and *K*. *marxianus* induced elevated levels of CD86 expression. Further, both yeasts induced significant down-regulation of CCR6 and up-regulation of CCR7, indicative of activation of immature DCs to a mature phenotype primed for lymph node migration and efficient antigen presentation.

### *K*. *marxianus* induces a strong T_reg_ cell response whereas *S*. *boulardii* induces a T cell response comprised of IFNγ, IL-17, and IL-10

**Fig 2:**
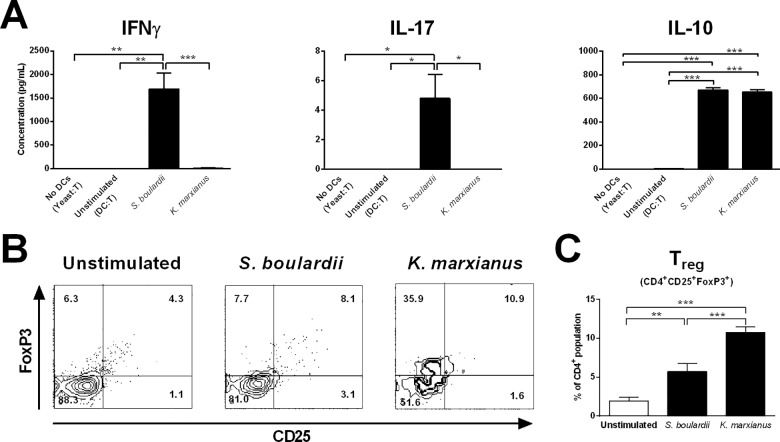
*K*. *marxianus* induces a strong T_reg_ cell response whereas *S*. *boulardii* induces a T cell response comprised of IFNγ, IL-17, and IL-10. T cell responses following 3 days co-incubation of yeast stimulated DCs and naive autologous T cells. For each yeast, DC stimulation was performed at a yeast:DC ratio of 10:1 (MOI 10), and the subsequent co-incubation of DCs and naive T cells was performed at a DC:T cell ratio of 1:20. **A.** T cell secretion levels of IFNγ, IL-17, and IL-10. Data are representative of at least five independent experiments, error bars represent SEM. **B.** Quantification of the T_reg_ subset as CD4^+^CD25^+^Foxp3^+^ cells. Dotplots gated on the CD4^+^ cell population from a single representative donor displaying the percentage of cells in each quadrant. **C.** Data from three independent experiments (three donors) expressed as the percentage of CD25^+^Foxp3^+^ cells in the CD4^+^ cell population, error bars represent SEM. One-way ANOVA, Tukey’s multiple comparisons test, indicating significant differences as follows: *, P<0.05; **, P<0.01; ***, P<0.001; ns, not significant.

Given that a key mechanism behind microbial modulation of excessive intestinal inflammation involves induction of immune responses driven by T_reg_ cells, we evaluated the ability of *K*. *marxianus* or *S*. *boulardii* to induce DC modulation of T cell function *in vitro*. We co-incubated yeast exposed DCs with autologous, naive T cells for 3 days, after which secreted levels of IFNγ, IL-17, and IL-10 were quantified. This co-incubation time was chosen based on time-course data showing equivalent levels of cytokine secretion following co-incubation for 3, 5, and 7 days (data not shown). As presented in Fig 2A, DCs exposed to the probiotic reference strain *S*. *boulardii* induced high levels of T cell IFNγ secretion, low but detectable IL-17 secretion, and robust IL-10 secretion, suggesting a *S*. *boulardii* induced T cell response composed of IFNγ secreting T_H_1 cells, IL-17 secreting T_H_17 cells, as well as IL-10 secreting T_reg_ cells. In contrast, despite secreting robust levels of IL-12 (Fig 1A), DCs exposed to *K*. *marxianus* induced no detectable T cell secretion of IFNγ or IL-17, but robust IL-10 secretion (Fig 2A), indicating a *K*. *marxianus* induced T cell response dominated by IL-10 secreting T_reg_ cells. In control wells containing yeast cells and naive T cells, but no DCs, we observed no cytokine secretion (Fig 2A), indicating that any detected cytokine secretion resulted from interactions between yeast stimulated DCs and naive T cells rather than direct yeast stimulation of T cells. Quantification of the T_reg_ subset by cellular immunostaining for CD4, CD25, and Foxp3 following 3 days co-incubation with yeast exposed DCs supported the cytokine data (Fig 2B and 2C). Whereas DCs exposed to *S*. *boulardii* induced elevated numbers of CD25^+^Foxp3^+^ cells compared to unstimulated cells, *K*. *marxianus* exposed DCs induced higher numbers of Foxp3^+^ cells and a greater expansion of the proportion of CD25^+^Foxp3^+^ cells.

### Yeast induced DC stimulation involves Dectin-1

**Fig 3:**
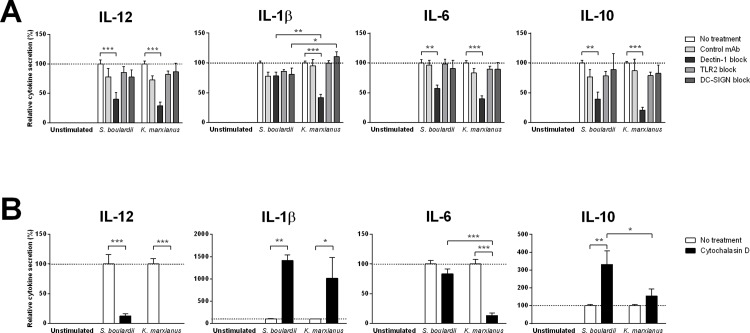
Yeast induced DC cytokine secretion relies on Dectin-1 mediated phagocytosis. DC secretion levels of IL-12, IL-1β, IL-6, and IL-10 following 20 h stimulation with DC media (unstimulated), a probiotic reference strain (*S*. *boulardii*), or *K*. *marxianus* at a yeast:DC ratio of 10:1 (MOI 10). **A.** For each yeast strain, DC stimulation was performed following a 30 min DC pre-incubation with HBSS (no treatment), a nonspecific isotype matched control antibody (control mAb) or monoclonal blocking antibodies specific for Dectin-1, TLR2, or DC-SIGN. Data are expressed as percent cytokine secretion relative to pre-incubation with HBSS (no treatment) and displayed as mean ± SEM of four independent experiments (four donors). **B.** For each yeast strain, DC stimulation was performed following a 30 min DC pre-incubation with HBSS (no treatment) or 20μM Cytochalasin D, a potent inhibitor of actin polymerization. Data are expressed as percent cytokine secretion relative to pre-incubation with HBSS (no treatment) and displayed as mean ± SEM of three independent experiments (three donors). Two-way ANOVA, Tukey’s multiple comparisons test. *, P<0.05; **, P<0.01; ***, P<0.001.

Using monoclonal blocking antibodies against the PRRs Dectin-1, TLR2, and DC-SIGN, we evaluated PRR involvement in yeast induced DC cytokine secretion. As displayed in Fig 3A, specific blockade of the β-glucan receptor Dectin-1 resulted in significantly lower levels of *K*. *marxianus* or *S*. *boulardii* induced DC cytokine secretion across all cytokines measured. In contrast, blocking TLR2 or DC-SIGN had no impact on yeast induced cytokine secretion.

As Dectin-1 is involved in initiating phagocytosis in myeloid phagocytes, we investigated the importance of yeast uptake for the observed yeast induced DC cytokine secretion. Obstructing phagocytosis through DC pretreatment with the specific inhibitor of actin polymerization cytochalasin D[36], we observed dramatic and opposing effects on yeast induced DC cytokine secretion (Fig 3B). Whereas DC pretreatment with cytochalasin D prevented yeast induced IL-12 secretion and *K*. *marxianus* induced IL-6 secretion, the opposite was true for yeast induced IL-1β and IL-10 secretion. Cytochalasin D pretreatment resulted in 10-fold higher levels of yeast induced IL-1β secretion and a clear upward trend in levels of yeast induced IL-10 secretion.

### Beta-glucan containing yeast cell wall extracts are potent inducers of IL-1β, IL-6, and IL-10 but fail to induce IL-12

**Fig 4:**
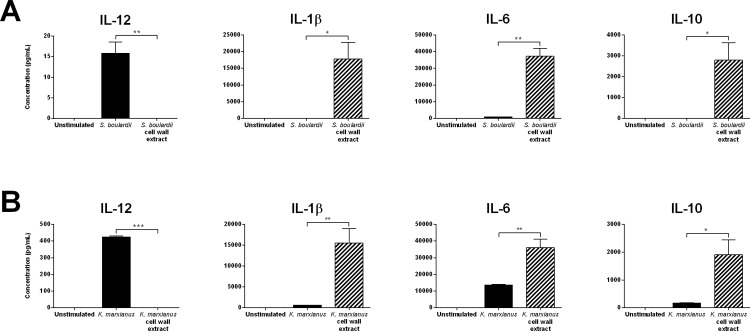
β-glucan containing yeast cell wall extracts are poor IL-12 inducers but potent inducers of DC IL-1β, IL-6, and IL-10 secretion. **A.** DC secretion levels of IL-12, IL-1β, IL-6, and IL-10 following 20 h stimulation with live *S*. *boulardii* or *S*. *boulardii* cell wall extract corresponding to a yeast:DC ratio of 10:1 (MOI 10). **B.** DC secretion levels of IL-12, IL-1β, IL-6, and IL-10 following 20 h stimulation with live *K*. *marxianus* or *K*. *marxianus* cell wall extract corresponding to a yeast:DC ratio of 10:1 (MOI 10). Data are representative of three independent experiments, error bars represent SEM. One-way ANOVA, Tukey’s multiple comparisons test. *, P<0.05; **, P<0.01; ***, P<0.001.

**Fig 5:**
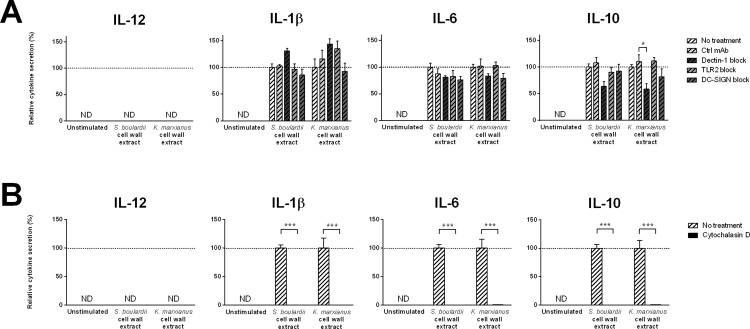
Yeast cell wall extract induced cytokine secretion involves multiple PRRs and is entirely dependent on DC uptake. DC secretion levels of IL-12, IL-1β, IL-6, and IL-10 following 20 h stimulation with DC media (unstimulated) or yeast cell wall extracts prepared from a probiotic reference strain (*S*. *boulardii*) or *K*. *marxianus* corresponding to a yeast:DC ratio of 10:1 (MOI 10). **A.** DC stimulation was performed following a 30 min DC pre-incubation with HBSS (no treatment), a nonspecific isotype matched control antibody (control mAb) or monoclonal blocking antibodies specific for Dectin-1, TLR2, or DC-SIGN. Data are expressed as percent cytokine secretion relative to pre-incubation with HBSS (no treatment) and displayed as mean ± SEM of two independent experiments. Two-way ANOVA, Dunnett’s multiple comparisons test. *, P<0.05; **, P<0.01; ***, P<0.001. ND, not detected. **B.** DC stimulation was performed following a 30 min DC pre-incubation with HBSS (no treatment) or 20μM Cytochalasin D, a potent inhibitor of actin polymerization. Data are expressed as percent cytokine secretion relative to pre-incubation with HBSS (no treatment) and displayed as mean ± SEM of two independent experiments. Two-way ANOVA, Tukey’s multiple comparisons test. *, P<0.05; **, P<0.01; ***, P<0.001. ND, not detected.

Based on the observation that Dectin-1, which recognizes fungal β-glucans, appeared to mediate yeast recognition in our experimental setup, we prepared β-glucan containing yeast cell wall extracts of *K*. *marxianus* and *S*. *boulardii* and explored their cytokine inducing properties in human monocyte-derived DCs (Fig 4). Strikingly, cell wall extracts prepared from either yeast strain induced DC secretion of IL-1β, IL-6, and IL-10 at levels significantly higher than observed with intact yeast cells. In contrast, none of the yeast cell wall extracts induced detectable levels of IL-12 secretion in DCs. As presented in Fig 5A, Dectin-1 blockade reduced the levels of DC IL-10 secretion induced by either yeast cell wall extract by approximately 40%, while DC IL-1β secretion levels appeared slightly elevated (by approximately 35%). Yeast cell wall extract induced DC IL-6 secretion appeared unaffected by Dectin-1 blockade, and in addition, blocking TLR2 or DC-SIGN had no significant effect on DC cytokine secretion induced by either cell wall extract (Fig 5A).

When investigating the importance of DC phagocytosis for yeast cell wall extract induced DC cytokine secretion, we found that blocking uptake through pretreatment with cytochalasin D completely obstructed cell wall extract induced DC cytokine secretion (Fig 5B). This contrasts with the observation that viable *K*. *marxianus* and *S*. *boulardii* were capable of inducing IL-1β and IL-10 secretion in DCs independently of phagocytic internalization (Fig 3B).

## Discussion

Although the number of fungal species is lower than the number of prokaryotic species present in the human gastrointestinal tract, the mycobiome rivals the microbiome in terms of biomass, indicating that fungi may play a bigger role in microbial communities than would be suspected from species numbers alone. In this context, commensal fungi and food-derived yeasts are known to interact with specialized immune cells present in the tissues lining the human gut. In the present study, a *S*. *boulardii* strain with probiotic effects documented in clinical trials[6] was found to modulate the function of human immune cells *in vitro*. This corresponds well with several studies by us and others reporting *S*. *boulardii* induced modulation of immune cell function *in vitro*[10, 37–39] and reduced inflammatory scores in experimental colitis models in rodents[32, 40–47]. Collectively, these findings indicate that *S*. *boulardii* administration has a beneficial impact on immune responses underlying intestinal inflammation. For the non-*Saccharomyces* yeast *K*. *marxianus*, typically isolated from fermented dairy products known for their health-promoting effects, we also observed modulation of human DC function *in vitro*. *K*. *marxianus* induced robust DC cytokine secretion across all cytokines measured, and this observed ability to modulate cytokine secretion of specialized immune cells agrees well with published reports[9, 10]. In addition, *K*. *marxianus* induced elevated expression levels of the co-stimulatory molecule CD86 but not CD80, and *K*. *marxianus* modulated DC chemokine receptor expression to levels near-identical to those induced by *S*. *boulardii*. We cannot exclude that the differential expression of CD80 and CD86 after stimulation with these two yeasts determines the resulting distinct T cell responses, as it has previously been reported that the relative effect of CD80 and CD86 influences T_H_ polarization and that CD80 blockade downregulates the T_H_1 response[48]. In addition, while CD80 and CD86 have been reported to play distinct functional roles in animal models of autoimmune disease, their individual contributions to shaping downstream T cell responses remain incompletely understood[49].

Collectively, these data demonstrate *K*. *marxianus* induced maturation of human DCs indicating functional similarities between *K*. *marxianus* and *S*. *boulardii*.

Subsequent co-incubation of yeast stimulated DCs and naive T cells revealed distinct T cell responses induced by the two yeasts. Whereas the established probiotic yeast *S*. *boulardii* induced a complex response involving IFNγ secreting T_H_1 cells as well as robust IL-10 secretion and increased numbers of Foxp3^+^ T_reg_ cells, *K*. *marxianus* induced a response dominated by IL-10 secretion and Foxp3^+^ T_reg_ cells. As numerous studies have linked microbial induction of Foxp3^+^ T_reg_ responses to beneficial effects in conditions characterized by excessive intestinal inflammation[20, 23–27, 50–52], this observation suggests that *K*. *marxianus* induced T_reg_ responses may contribute to mucosal tolerance *in vivo*.

Dectin-1 has emerged as a central PRR relating innate fungal recognition to human health, as observed by the fact that Dectin-1 deficiency results in an elevated susceptibility to colitis[4]. We identified Dectin-1 as key for DC recognition of *K*. *marxianus* as well as *S*. *boulardii* in our experimental setup, indicating that recognition relies on conserved β-glucans present in cell walls of yeasts representing phylogenetically distinct genera. This agrees well with a study on DC recognition and uptake of yeasts representing the *Saccharomyces*, *Kluyveromyces*, *Pichia*, and *Schizosaccharomyces* genera, in which Dectin-1 blockade resulted in significantly lower levels of phagocytic internalization of yeasts[53]. In contrast to reports of TLR2 involvement in DC recognition of the *S*. *cerevisiae* derived cell wall preparation zymosan[11], TLR2 blockade had no effect on yeast induced DC cytokine secretion in the present study. Similarly, the PRR DC-SIGN, which binds mannose- or fucose-containing carbohydrates in microbial cell walls, did not appear involved in DC recognition of *K*. *marxianus* or *S*. *boulardii* in our experimental setup. However, since Dectin-1 blockade did not entirely prevent yeast induced DC cytokine secretion (reduction to <50% of unobstructed conditions), additional PRRs are likely to contribute to yeast recognition. Whereas several studies have shown the mannose receptor to be involved in DC internalization of pathogenic yeasts such as *Candida albicans* and *Cryptococcus neoformans*[54, 55], a more recent study showed no modulation of *C*. *albicans* induced cytokine secretion by human PBMCs in the presence of a specific mannose receptor inhibitor[56].

In addition to induction of downstream signaling pathways leading to modulation of DC cytokine secretion, yeast recognition by Dectin-1 initiates phagocytic uptake[57]. In the present study, interfering with DC phagocytosis through cytochalasin D pretreatment had a significant impact on yeast induced DC cytokine secretion. *K*. *marxianus* induced DC IL-12 and IL-6 secretion were obstructed by cytochalasin D, indicating a mechanism dependent on phagocytic internalization of *K*. *marxianus*. In contrast, the significant increase in DC IL-1β secretion induced by *K*. *marxianus* or *S*. *boulardii* in the presence of cytochalasin D indicates a mechanism operating independently of yeast uptake. While the observed effects of cytochalasin D pretreatment appear to reflect inhibition of phagocytic uptake of the yeasts, this may be more conclusively proven by including a non-particulate stimulus in the experimental setup, something that was not part of the present study.

Interestingly, Dectin-1 mediated activation of a noncanonical caspase-8 inflammasome capable of both induction and maturation of IL-1β independently of microbial internalization was described recently[58], a finding that may support our observation of yeast induced DC IL-1β secretion in the absence of phagocytic uptake. Specifically, *C*. *albicans* was found to induce induction and maturation of IL-1β in human DCs through a mechanism dependent upon Dectin-1, ASC, and caspase-8 but independently of NLPR3, caspase-1, and phagocytosis[58]. However, alternative IL-1β processing via caspase-8 was not experimentally confirmed in the present study.

In the context of immune modulating properties of yeasts, it is well established that fungal β-glucans have immune potentiating effects[28, 29]. In the present study, DC stimulation with β-glucan containing yeast cell wall extracts prepared from *K*. *marxianus* or *S*. *boulardii* elicited significantly higher secretion levels of IL-1β, IL-6, and IL-10 as compared to stimulation with intact yeast cells. As β-glucan masking by outer cell wall layers of mannan or α-glucans is an established immune evasion strategy employed by fungal pathogens[59–61], and as the yeast cell wall extract preparation procedure performed in the present study was designed for β-glucan extraction, demasking of β-glucans is likely a contributing factor in the observed immune potentiation compared to intact yeast cells. Whereas live *K*. *marxianus* did not induce significant levels of T cell IL-17 secretion, on the basis of the data presented in this study, it cannot be excluded that the significantly elevated levels of DC secretion of IL-1β and IL-6 induced by stimulation with *K*. *marxianus* cell wall extract may result in T cell IL-17 secretion. Future studies are needed to clarify this.

Strikingly, cell wall extracts prepared from *K*. *marxianus* or *S*. *boulardii* failed to induce detectable levels of DC IL-12 secretion in the present study. This mirrors findings from a study comparing cytokine inducing properties of β-glucans from numerous sources, in which yeast-derived β-glucans induced IL-6 and IL-10 but no detectable IL-12[62]. A similar discrepancy between IL-12 inducing properties of intact cells and isolated cell wall components has been reported for DC stimulation with lactobacilli[35]. Thus, as synergistic signaling involving multiple PRRs may be required for certain cytokine responses[63], the lack of detectable DC IL-12 secretion induced by yeast cell wall extracts in our experimental setup may indicate that Dectin-1 signaling alone is insufficient for generation of an IL-12 response. In the present study, IL-12 secretion induced by intact *K*. *marxianus* or *S*. *boulardii* cells was primarily dependent on Dectin-1 and entirely uptake-dependent. Collectively, our observations support a mechanism for IL-12 induction based on Dectin-1 mediated DC uptake of intact yeast cells, PRR clustering at the phagocytic synapse, and a necessity for additional PRR signaling resulting in DC secretion of IL-12.

In the present study, the immune modulating properties of *K*. *marxianus* were characterized alongside *S*. *boulardii* in an effort to evaluate whether *K*. *marxianus* expresses properties consistent with probiotic functionality. Importantly, in order to truly assess the suitability of *K*. *marxianus* as a probiotic yeast, further studies will need to include comparative testing against a pathogenic species such as *C*. *albicans*. In conclusion, the present study demonstrates distinct levels of DC cytokine secretion and significant differences in T cell responses induced by *K*. *marxianus* and *S*. *boulardii*. For both yeasts, the observed interactions with human DCs appeared to be primarily driven by Dectin-1 recognition of β-glucan components in their cell walls. Specifically, β-glucan containing yeast cell wall extracts induced potent DC secretion of IL-1β, IL-6, and IL-10, whereas IL-12 induction relied on Dectin-1 mediated uptake of intact yeast cells, indicating the involvement of multiple PRRs. Upon co-incubation of yeast exposed DCs and naive T cells, *S*. *boulardii* induced a potent IFNγ response indicating T_H_1 mobilization. In contrast, *K*. *marxianus* induced a response dominated by Foxp3^+^ T_reg_ cells, a characteristic that may benefit human health in conditions characterized by excessive inflammation and positions *K*. *marxianus* as a strong candidate for further development as a novel yeast probiotic.

## References

[1] Clemente JC, Ursell LK, Parfrey LW, Knight R. The impact of the gut microbiota on human health: an integrative view. Cell. 2012;148(6):1258–70. 10.1016/j.cell.2012.01.035 22424233 PMC5050011

[2] Coombes JL, Powrie F. Dendritic cells in intestinal immune regulation. Nat Rev Immunol. 2008;8(6):435–46. PubMed Central PMCID: PMC2674208. 10.1038/nri2335 18500229 PMC2674208

[3] Marchesi JR. Prokaryotic and eukaryotic diversity of the human gut. Adv Appl Microbiol. 2010;72:43–62. 10.1016/S0065-2164(10)72002-5 20602987

[4] Iliev ID, Funari VA, Taylor KD, Nguyen Q, Reyes CN, Strom SP, et al Interactions between commensal fungi and the C-type lectin receptor Dectin-1 influence colitis. Science. 2012;336(6086):1314–7. PubMed Central PMCID: PMC3432565. 10.1126/science.1221789 22674328 PMC3432565

[5] van der Aa Kuhle A, Jespersen L. The taxonomic position of Saccharomyces boulardii as evaluated by sequence analysis of the D1/D2 domain of 26S rDNA, the ITS1-5.8S rDNA-ITS2 region and the mitochondrial cytochrome-c oxidase II gene. Syst Appl Microbiol. 2003;26(4):564–71. 10.1078/072320203770865873 14666985

[6] McFarland LV. Systematic review and meta-analysis of Saccharomyces boulardii in adult patients. World J Gastroenterol. 2010;16(18):2202–22. PubMed Central PMCID: PMC2868213. 10.3748/wjg.v16.i18.2202 20458757 PMC2868213

[7] Kumura H, Tanoue Y, Tsukahara M, Tanaka T, Shimazaki K. Screening of dairy yeast strains for probiotic applications. J Dairy Sci. 2004;87(12):4050–6. 10.3168/jds.S0022-0302(04)73546-8 15545365

[8] (BIOHAZ) EPoBH. Scientific Opinion on the maintenance of the list of QPS agents intentionally added to food and feed (2010 update). EFSA Journal. 2010;8(12).

[9] Maccaferri S, Klinder A, Brigidi P, Cavina P, Costabile A. Potential probiotic Kluyveromyces marxianus B0399 modulates the immune response in Caco-2 cells and peripheral blood mononuclear cells and impacts the human gut microbiota in an in vitro colonic model system. Appl Environ Microbiol. 2012;78(4):956–64. PubMed Central PMCID: PMC3272993. 10.1128/AEM.06385-11 22156412 PMC3272993

[10] Smith IM, Christensen JE, Arneborg N, Jespersen L. Yeast modulation of human dendritic cell cytokine secretion: an in vitro study. PLoS One. 2014;9(5):e96595 PubMed Central PMCID: PMC4015989. 10.1371/journal.pone.0096595 24816850 PMC4015989

[11] Dillon S, Agrawal S, Banerjee K, Letterio J, Denning TL, Oswald-Richter K, et al Yeast zymosan, a stimulus for TLR2 and dectin-1, induces regulatory antigen-presenting cells and immunological tolerance. J Clin Invest. 2006;116(4):916–28. PubMed Central PMCID: PMC1401484. 10.1172/JCI27203 16543948 PMC1401484

[12] Kawai T, Akira S. Toll-like receptors and their crosstalk with other innate receptors in infection and immunity. Immunity. 2011;34(5):637–50. 10.1016/j.immuni.2011.05.006 21616434

[13] Hardison SE, Brown GD. C-type lectin receptors orchestrate antifungal immunity. Nat Immunol. 2012;13(9):817–22. PubMed Central PMCID: PMC3432564. 10.1038/ni.2369 22910394 PMC3432564

[14] Geijtenbeek TB, Torensma R, van Vliet SJ, van Duijnhoven GC, Adema GJ, van Kooyk Y, et al Identification of DC-SIGN, a novel dendritic cell-specific ICAM-3 receptor that supports primary immune responses. Cell. 2000;100(5):575–85. 10721994 10.1016/s0092-8674(00)80693-5

[15] Brown GD, Gordon S. Immune recognition of fungal beta-glucans. Cell Microbiol. 2005;7(4):471–9. 10.1111/j.1462-5822.2005.00505.x 15760447

[16] Brown GD. Dectin-1: a signalling non-TLR pattern-recognition receptor. Nat Rev Immunol. 2006;6(1):33–43. 10.1038/nri1745 16341139

[17] Donkor ON, Ravikumar M, Proudfoot O, Day SL, Apostolopoulos V, Paukovics G, et al Cytokine profile and induction of T helper type 17 and regulatory T cells by human peripheral mononuclear cells after microbial exposure. Clin Exp Immunol. 2012;167(2):282–95. PubMed Central PMCID: PMC3278695. 10.1111/j.1365-2249.2011.04496.x 22236005 PMC3278695

[18] Maynard CL, Elson CO, Hatton RD, Weaver CT. Reciprocal interactions of the intestinal microbiota and immune system. Nature. 2012;489(7415):231–41. PubMed Central PMCID: PMC4492337. 10.1038/nature11551 22972296 PMC4492337

[19] Hooper LV, Littman DR, Macpherson AJ. Interactions between the microbiota and the immune system. Science. 2012;336(6086):1268–73. PubMed Central PMCID: PMC4420145. 10.1126/science.1223490 22674334 PMC4420145

[20] Kwon HK, Lee CG, So JS, Chae CS, Hwang JS, Sahoo A, et al Generation of regulatory dendritic cells and CD4+Foxp3+ T cells by probiotics administration suppresses immune disorders. Proc Natl Acad Sci U S A. 2010;107(5):2159–64. PubMed Central PMCID: PMC2836639. 10.1073/pnas.0904055107 20080669 PMC2836639

[21] Liu Y, Tran DQ, Fatheree NY, Marc Rhoads J. Lactobacillus reuteri DSM 17938 differentially modulates effector memory T cells and Foxp3+ regulatory T cells in a mouse model of necrotizing enterocolitis. Am J Physiol Gastrointest Liver Physiol. 2014;307(2):G177–86. PubMed Central PMCID: PMC4101683. 10.1152/ajpgi.00038.2014 24852566 PMC4101683

[22] Smelt MJ, de Haan BJ, Bron PA, van Swam I, Meijerink M, Wells JM, et al L. plantarum, L. salivarius, and L. lactis attenuate Th2 responses and increase Treg frequencies in healthy mice in a strain dependent manner. PLoS One. 2012;7(10):e47244 PubMed Central PMCID: PMC3467239. 10.1371/journal.pone.0047244 23056616 PMC3467239

[23] Bassaganya-Riera J, Viladomiu M, Pedragosa M, De Simone C, Hontecillas R. Immunoregulatory mechanisms underlying prevention of colitis-associated colorectal cancer by probiotic bacteria. PLoS One. 2012;7(4):e34676 PubMed Central PMCID: PMC3325233. 10.1371/journal.pone.0034676 22511958 PMC3325233

[24] Petersen ER, Claesson MH, Schmidt EG, Jensen SS, Ravn P, Olsen J, et al Consumption of probiotics increases the effect of regulatory T cells in transfer colitis. Inflamm Bowel Dis. 2012;18(1):131–42. 10.1002/ibd.21709 21495121

[25] Zheng B, van Bergenhenegouwen J, Overbeek S, van de Kant HJ, Garssen J, Folkerts G, et al Bifidobacterium breve attenuates murine dextran sodium sulfate-induced colitis and increases regulatory T cell responses. PLoS One. 2014;9(5):e95441 PubMed Central PMCID: PMC4008378. 10.1371/journal.pone.0095441 24787575 PMC4008378

[26] Qiu X, Zhang M, Yang X, Hong N, Yu C. Faecalibacterium prausnitzii upregulates regulatory T cells and anti-inflammatory cytokines in treating TNBS-induced colitis. J Crohns Colitis. 2013;7(11):e558–68. 10.1016/j.crohns.2013.04.002 23643066

[27] Tanoue T, Honda K. Induction of Treg cells in the mouse colonic mucosa: a central mechanism to maintain host-microbiota homeostasis. Semin Immunol. 2012;24(1):50–7. 10.1016/j.smim.2011.11.009 22172550

[28] Seong SK, Kim HW. Potentiation of Innate Immunity by beta-Glucans. Mycobiology. 2010;38(2):144–8. PubMed Central PMCID: PMC3741566. 10.4489/MYCO.2010.38.2.144 23956643 PMC3741566

[29] Batbayar S, Lee DH, Kim HW. Immunomodulation of Fungal beta-Glucan in Host Defense Signaling by Dectin-1. Biomol Ther (Seoul). 2012;20(5):433–45. PubMed Central PMCID: PMC3762275. 24009832 10.4062/biomolther.2012.20.5.433 PMC3762275

[30] Qi C, Cai Y, Gunn L, Ding C, Li B, Kloecker G, et al Differential pathways regulating innate and adaptive antitumor immune responses by particulate and soluble yeast-derived beta-glucans. Blood. 2011;117(25):6825–36. PubMed Central PMCID: PMC3128477. 10.1182/blood-2011-02-339812 21531981 PMC3128477

[31] Karumuthil-Melethil S, Gudi R, Johnson BM, Perez N, Vasu C. Fungal beta-glucan, a Dectin-1 ligand, promotes protection from type 1 diabetes by inducing regulatory innate immune response. J Immunol. 2014;193(7):3308–21. PubMed Central PMCID: PMC4170060. 10.4049/jimmunol.1400186 25143443 PMC4170060

[32] Jawhara S, Habib K, Maggiotto F, Pignede G, Vandekerckove P, Maes E, et al Modulation of intestinal inflammation by yeasts and cell wall extracts: strain dependence and unexpected anti-inflammatory role of glucan fractions. PLoS One. 2012;7(7):e40648 PubMed Central PMCID: PMC3407157. 10.1371/journal.pone.0040648 22848391 PMC3407157

[33] Jespersen L, Nielsen DS, Hønholt S, Jakobsen M. Occurrence and diversity of yeasts involved in fermentation of West African cocoa beans. FEMS Yeast Research. 2005;5(4–5):441–53. 10.1016/j.femsyr.2004.11.002 15691749

[34] de Groot PW, de Boer AD, Cunningham J, Dekker HL, de Jong L, Hellingwerf KJ, et al Proteomic analysis of Candida albicans cell walls reveals covalently bound carbohydrate-active enzymes and adhesins. Eukaryot Cell. 2004;3(4):955–65. PubMed Central PMCID: PMC500891. 10.1128/EC.3.4.955-965.2004 15302828 PMC500891

[35] Zeuthen LH, Fink LN, Frokiaer H. Toll-like receptor 2 and nucleotide-binding oligomerization domain-2 play divergent roles in the recognition of gut-derived lactobacilli and bifidobacteria in dendritic cells. Immunology. 2008;124(4):489–502. PubMed Central PMCID: PMC2492941. 10.1111/j.1365-2567.2007.02800.x 18217947 PMC2492941

[36] Goddette DW, Frieden C. Actin polymerization. The mechanism of action of cytochalasin D. J Biol Chem. 1986;261(34):15974–80. 3023337

[37] Thomas S, Metzke D, Schmitz J, Dorffel Y, Baumgart DC. Anti-inflammatory effects of Saccharomyces boulardii mediated by myeloid dendritic cells from patients with Crohn's disease and ulcerative colitis. Am J Physiol Gastrointest Liver Physiol. 2011;301(6):G1083–92. 10.1152/ajpgi.00217.2011 21903765

[38] Thomas S, Przesdzing I, Metzke D, Schmitz J, Radbruch A, Baumgart DC. Saccharomyces boulardii inhibits lipopolysaccharide-induced activation of human dendritic cells and T cell proliferation. Clin Exp Immunol. 2009;156(1):78–87. PubMed Central PMCID: PMC2673744. 10.1111/j.1365-2249.2009.03878.x 19161443 PMC2673744

[39] Gad M, Ravn P, Soborg DA, Lund-Jensen K, Ouwehand AC, Jensen SS. Regulation of the IL-10/IL-12 axis in human dendritic cells with probiotic bacteria. FEMS Immunol Med Microbiol. 2011;63(1):93–107. 10.1111/j.1574-695X.2011.00835.x 21707779

[40] Martins FS, Dalmasso G, Arantes RM, Doye A, Lemichez E, Lagadec P, et al Interaction of Saccharomyces boulardii with Salmonella enterica serovar Typhimurium protects mice and modifies T84 cell response to the infection. PLoS One. 2010;5(1):e8925 PubMed Central PMCID: PMC2811747. 10.1371/journal.pone.0008925 20111723 PMC2811747

[41] Martins FS, Nardi RM, Arantes RM, Rosa CA, Neves MJ, Nicoli JR. Screening of yeasts as probiotic based on capacities to colonize the gastrointestinal tract and to protect against enteropathogen challenge in mice. J Gen Appl Microbiol. 2005;51(2):83–92. 15942869 10.2323/jgam.51.83

[42] Dalmasso G, Cottrez F, Imbert V, Lagadec P, Peyron JF, Rampal P, et al Saccharomyces boulardii inhibits inflammatory bowel disease by trapping T cells in mesenteric lymph nodes. Gastroenterology. 2006;131(6):1812–25. 10.1053/j.gastro.2006.10.001 17087945

[43] Lee SK, Kim YW, Chi SG, Joo YS, Kim HJ. The effect of Saccharomyces boulardii on human colon cells and inflammation in rats with trinitrobenzene sulfonic acid-induced colitis. Dig Dis Sci. 2009;54(2):255–63. 10.1007/s10620-008-0357-0 18612822

[44] Foligne B, Dewulf J, Vandekerckove P, Pignede G, Pot B. Probiotic yeasts: anti-inflammatory potential of various non-pathogenic strains in experimental colitis in mice. World J Gastroenterol. 2010;16(17):2134–45. PubMed Central PMCID: PMC2864839. 10.3748/wjg.v16.i17.2134 20440854 PMC2864839

[45] Chen X, Kokkotou EG, Mustafa N, Bhaskar KR, Sougioultzis S, O'Brien M, et al Saccharomyces boulardii inhibits ERK1/2 mitogen-activated protein kinase activation both in vitro and in vivo and protects against Clostridium difficile toxin A-induced enteritis. J Biol Chem. 2006;281(34):24449–54. 10.1074/jbc.M605200200 16816386

[46] Justino PF, Melo LF, Nogueira AF, Costa JV, Silva LM, Santos CM, et al Treatment with Saccharomyces boulardii reduces the inflammation and dysfunction of the gastrointestinal tract in 5-fluorouracil-induced intestinal mucositis in mice. Br J Nutr. 2014;111(9):1611–21. 10.1017/S0007114513004248 24503021

[47] Everard A, Matamoros S, Geurts L, Delzenne NM, Cani PD. Saccharomyces boulardii administration changes gut microbiota and reduces hepatic steatosis, low-grade inflammation, and fat mass in obese and type 2 diabetic db/db mice. MBio. 2014;5(3):e01011–14. PubMed Central PMCID: PMC4056549. 10.1128/mBio.01011-14 24917595 PMC4056549

[48] Lang TJ, Nguyen P, Peach R, Gause WC, Via CS. In vivo CD86 blockade inhibits CD4+ T cell activation, whereas CD80 blockade potentiates CD8+ T cell activation and CTL effector function. J Immunol. 2002;168(8):3786–92. 11937530 10.4049/jimmunol.168.8.3786

[49] Podojil JR, Miller SD. Molecular mechanisms of T-cell receptor and costimulatory molecule ligation/blockade in autoimmune disease therapy. Immunological Reviews. 2009;229(1):337–55. 10.1111/j.1600-065X.2009.00773.x 19426232 PMC2845642

[50] Atarashi K, Tanoue T, Oshima K, Suda W, Nagano Y, Nishikawa H, et al Treg induction by a rationally selected mixture of Clostridia strains from the human microbiota. Nature. 2013;500(7461):232–6. 10.1038/nature12331 23842501

[51] Round JL, Mazmanian SK. Inducible Foxp3+ regulatory T-cell development by a commensal bacterium of the intestinal microbiota. Proc Natl Acad Sci U S A. 2010;107(27):12204–9. PubMed Central PMCID: PMC2901479. 10.1073/pnas.0909122107 20566854 PMC2901479

[52] Konieczna P, Groeger D, Ziegler M, Frei R, Ferstl R, Shanahan F, et al Bifidobacterium infantis 35624 administration induces Foxp3 T regulatory cells in human peripheral blood: potential role for myeloid and plasmacytoid dendritic cells. Gut. 2012;61(3):354–66. 10.1136/gutjnl-2011-300936 22052061

[53] Bazan SB, Geginat G, Breinig T, Schmitt MJ, Breinig F. Uptake of various yeast genera by antigen-presenting cells and influence of subcellular antigen localization on the activation of ovalbumin-specific CD8 T lymphocytes. Vaccine. 2011;29(45):8165–73. 10.1016/j.vaccine.2011.07.141 21856351

[54] Romani L, Montagnoli C, Bozza S, Perruccio K, Spreca A, Allavena P, et al The exploitation of distinct recognition receptors in dendritic cells determines the full range of host immune relationships with Candida albicans. Int Immunol. 2004;16(1):149–61. 14688070 10.1093/intimm/dxh012

[55] Mansour MK, Latz E, Levitz SM. Cryptococcus neoformans glycoantigens are captured by multiple lectin receptors and presented by dendritic cells. J Immunol. 2006;176(5):3053–61. 16493064 10.4049/jimmunol.176.5.3053

[56] van de Veerdonk FL, Marijnissen RJ, Kullberg BJ, Koenen HJ, Cheng SC, Joosten I, et al The macrophage mannose receptor induces IL-17 in response to Candida albicans. Cell Host Microbe. 2009;5(4):329–40. 10.1016/j.chom.2009.02.006 19380112

[57] Kerrigan AM, Brown GD. C-type lectins and phagocytosis. Immunobiology. 2009;214(7):562–75. PubMed Central PMCID: PMC2702671. 10.1016/j.imbio.2008.11.003 19261355 PMC2702671

[58] Gringhuis SI, Kaptein TM, Wevers BA, Theelen B, van der Vlist M, Boekhout T, et al Dectin-1 is an extracellular pathogen sensor for the induction and processing of IL-1beta via a noncanonical caspase-8 inflammasome. Nat Immunol. 2012;13(3):246–54. 10.1038/ni.2222 22267217

[59] Gantner BN, Simmons RM, Underhill DM. Dectin-1 mediates macrophage recognition of Candida albicans yeast but not filaments. EMBO J. 2005;24(6):1277–86. PubMed Central PMCID: PMC556398. 10.1038/sj.emboj.7600594 15729357 PMC556398

[60] Bain JM, Louw J, Lewis LE, Okai B, Walls CA, Ballou ER, et al Candida albicans hypha formation and mannan masking of beta-glucan inhibit macrophage phagosome maturation. MBio. 2014;5(6):e01874 PubMed Central PMCID: PMC4324242. 10.1128/mBio.01874-14 25467440 PMC4324242

[61] Rappleye CA, Eissenberg LG, Goldman WE. Histoplasma capsulatum alpha-(1,3)-glucan blocks innate immune recognition by the beta-glucan receptor. Proc Natl Acad Sci U S A. 2007;104(4):1366–70. PubMed Central PMCID: PMC1783108. 10.1073/pnas.0609848104 17227865 PMC1783108

[62] Wismar R, Brix S, Laerke HN, Frokiaer H. Comparative analysis of a large panel of non-starch polysaccharides reveals structures with selective regulatory properties in dendritic cells. Mol Nutr Food Res. 2011;55(3):443–54. 10.1002/mnfr.201000230 20938988

[63] Gantner BN, Simmons RM, Canavera SJ, Akira S, Underhill DM. Collaborative induction of inflammatory responses by dectin-1 and Toll-like receptor 2. J Exp Med. 2003;197(9):1107–17. PubMed Central PMCID: PMC2193968. 10.1084/jem.20021787 12719479 PMC2193968

